# Quality of life in men with chronic scrotal pain

**DOI:** 10.1080/24740527.2017.1328592

**Published:** 2017-08-17

**Authors:** Aosama Aljumaily, Christopher Wu, Hind Al-Khazraji, Allan Gordon, Susan Lau, Keith A. Jarvi

**Affiliations:** a Division of Urology, Department of Surgery, Mount Sinai Hospital, University of Toronto, Toronto, Ontario, Canada; b Murray Koffler Urologic Wellness Centre, Mount Sinai Hospital, Toronto, Ontario, Canada; c Division of Urology, Department of Surgery, McMaster University, Hamilton, Ontario, Canada; d Wasser Pain Management Centre, Mount Sinai Hospital, Toronto, Ontario, Canada; e Lunenfeld-Tanenbaum Research Institute, Mount Sinai Hospital, Toronto, Ontario, Canada; f Institute of Medical Sciences, University of Toronto, Toronto, Ontario, Canada

**Keywords:** Chronic orchialgia, chronic scrotal pain, quality of life, questionnaires

## Abstract

**Background**: Chronic scrotal pain (CSP) is a common and often debilitating condition found in up to 4.75% of men. There is little written on the impact of CSP on men’s lives.

**Aim**: The aim of this study was to understand the impact of CSP on men’s lives.

**Methods**: Patients with CSP were prospectively asked to complete a comprehensive questionnaire, including questions on quality of life (QoL), activities, and mood.

**Results**: The mean age of the 131 patients was 43 years. Pain was intermittent, with severe pain episodes (mean pain scores 7.2 ± 2 out of 10) affecting men on average 40% ± 30% of the time. Overall, 88/131 (67.17%) of patients responded that they felt “unhappy” or “terrible” with their present situation. More than 40% of patients complained of depressive symptoms more than half the days of the month. Normal activities were adversely affected, with 68/131 (51.90%) reporting limited ability to work, 93/131 (70.99%) patients reporting decreased physical activity, and 81/131(61.83%) reporting decreased sexual activity. Comparing men with pain levels ≥ 7/10 vs. those with pain levels < 7/10, 47% (41/88) vs. 8.1% (3/37) reported that they felt “terrible,” 40% (35/88) vs. 13% (5/38) had depressive feelings more than half the time, and 35% (28/80) vs. 16% (6/38) felt little pleasure doing things (*P* < 0.01 for all).

**Conclusion**: Our study suggests that QoL, mood, and the ability to perform normal activities are profoundly disturbed in CSP patients and that the pain severity is directly related to QoL.

## RéSUMé

**Contexte:** La douleur scrotale chronique est une affection commune et souvent débilitante dont souffrent près de 4,75 % des hommes. Il y a peu d’écrits sur l’impact de la douleur scrotale chronique sur la vie des hommes.

**But:** Comprendre l’impact de la douleur scrotale chronique sur la vie des hommes.

**Méthodes:** On a demandé de manière prospective à des patients souffrant de douleur scrotale chronique de remplir un questionnaire détaillé comprenant des questions sur leur qualité de vie, leurs activités et leur humeur.

**Résultats:** L’âge moyen des 131 patients était de 43 ans. La douleur était intermittente, comportait des épisodes de douleur intense (scores de la douleur moyens 7,2 +/- 2 sur 10) et affectait les hommes 40 % +/- 30 % du temps. Globalement, 88/131 (67,17 %) des patients ont répondu qu’ils se sentaient malheureux ou « horriblement mal » en raison de leur situation actuelle. Plus de 40 % des patients se sont plaints de symptômes de dépression plus de la moitié des jours du mois. Leurs activités habituelles étaient affectées négativement, alors que 68/131 (51,90 %) disaient avoir une capacité de travail limitée, que 93/131 (70,99 %) des patients signalaient une diminution de leur activité physique et que 81/131 (61,83 %) d’entre eux faisaient état d’une diminution de leur activité sexuelle. La comparaison des hommes qui avaient un niveau de douleur ≥ 7/10 à ceux qui avaient un niveau de douleur < 7/10 a révélé que 47 % d’entre eux (41/88) se sentaient « horriblement pas bien » contre 8,1 % (3/37), que 40 % (35/88) avaient des sentiments dépressifs plus de la moitié du temps contre 13 % (5/38) et que 35 % (28/80) ressentaient peu de plaisir à faire les choses contre 16 % (6/38) (p < 0,01 pour tous).

**Conclusion:** Notre étude suggère que la qualité de vie, l’humeur et la capacité à vaquer à ses activités habituelles sont profondément perturbées chez les patients qui souffrent de douleur scrotale chronique et que l’intensité de la douleur est directement liée à la qualité de vie.

## Introduction

*Chronic scrotal pain* (CSP) refers to intermittent or constant testicular pain for a period of three or more months that interferes with daily activities.^1^ The pain may originate from any of the scrotal contents, including the testicle, epididymis, paratesticular structures, and spermatic cord or be referred from the groin (hernias or conjoint tendon injury) or retroperitoneum.^2^

Both the European Urological Association and the International Continence Society have adopted the generic term *scrotal pain syndrome* to include testicular pain syndrome, postvasectomy pain syndrome, and epididymal pain syndrome.^3–6^

CSP is a common problem that most pain specialists will encounter frequently during their careers. Though we understand that CSP is a common condition, there are few reports on the true incidence of this debilitating condition. Cifti et al. reported that 4.75% of men being assessed in a urology clinic for other conditions also had CSP.^7^ This is similar to our center’s data, where 4.3% of men presenting for investigation of infertility also complained of CSP (unpublished data). A lower incidence of CSP was estimated in a report from Switzerland, where, based on a survey of Swiss urologists who were asked to provide figures on the patients seen in their clinics with CSP, provided an estimate of an incidence of 350–400 cases of CSP per 100 000 men per year.^8^ In the United States, scrotal pain is the most common urological reason for medical discharge from the U.S. Army and was the most common urological complaint among enlisted men deployed to Iraq.^4,9^

This condition is often frustrating for clinicians, because many of the patients present with a debilitating condition and, more often than not, no known etiology for the CSP is found.^10^ Close to 50% of men presenting with CSP have an unknown cause for the pain. Compounding the complexity for the clinicians, chronic pain may lead to neuropathic changes, which may lead to neuropathic pain.^10^

Because clinicians often do not have answers or at least answers that satisfy some of the patients, men seek evaluation from multiple providers in an attempt to uncover an explanation and treatment for their CSP.^4^

Though there are few studies on the impact of CSP on men’s lives and activities, the effect seems to be significant, with limitations in ability to work, impacts on social activities, and negative effects on sexual function and activity.^11^ We also have a study indicating that psychological issues are common in men with chronic scrotal pain presenting with problems related to depression, anxiety, hysteria, hypochondriasis, and somatization disorders.^12^ These psychological stresses were felt to either contribute to or exacerbate CSP.

Despite the fact that quality of life (QoL) is known to be highly disturbed in CSP patients, there are no studies in the literature quantitatively evaluating the quality of life for men with CSP. The purpose of this study is to describe the QoL for men with CSP and the impact of CSP on men’s work and social lives.

## Materials and methods

Mount Sinai Hospital local ethical committee approval was obtained prior to commencement of the study. From February 2014 to September 2015, a total of 131 men presented for assessment of CSP. This assessment was performed at the Multidisciplinary Orchialgia Clinic located at Mount Sinai Hospital in Toronto, Canada. This clinic is a multidisciplinary clinic in which the patient is evaluated simultaneously by pain medicine specialists and by urologists with special expertise in CSP.

A detailed history and physical were obtained from all subjects. At the initial consultation, all patients were asked to complete a comprehensive questionnaire, which included date of birth, questions on the duration of the pain, a standardized detailed pain history including a validated numeric pain scale to quantify pain levels from one to ten,^13,14^ as well as standardized questions pertaining to the impact of CSP on QoL.

### Pain severity

The pain level was assessed with the two questions, “How bad was your most severe (worst) pain in last month?” and “When you have pain, how bad was your average pain in the last month?” graded from zero to ten.

### Effect of CSP on work performance, normal activities, and sexual activities

The impact of CSP on work performance was assessed using an internally developed question, “How much have your symptoms kept you from performing your normal work?” with the choice of answers: *none, only a little, some*, and *a lot*. Similarly, the impact of CSP on daily activities was assessed with the question, “How much have your symptoms kept you from doing the kinds of things you would usually do, over the last month?” with the same choices of answers as above. Finally, the effect of CSP on sexual activity was recorded with the question, “How much have your symptoms kept you from normal sexual activity?” with the same choice of answers.

### Depressive symptoms

The impact of CSP on depressive symptoms was assessed by two validated questions, “During the past month, have you often been bothered by feeling down, depressed, or hopeless?” and “During the past month, have you often been bothered by little interest or pleasure in doing things?”^15^ Scores were quantified by the answers *not at all, several days, more than half the days*, and *nearly every day* for both of these questions.^15^

If patients reported depressive symptoms, the patients were directly questioned about suicidal thoughts.

### Quality of life

Quality of life was assessed with the previously published question, “If you were to spend the rest of your life with your symptoms just the way they have been during the last month, how would you feel about that?” Scores for this question were quantified on a linear scale from one to seven as 1 = *delighted*, 2 = *pleased*, 3 = *mostly satisfied*, 4 = *mixed*, 5 = *mostly dissatisfied*, 6 = *unhappy*, and 7 = *terrible*.^14^

Earlier work by Andrews and Crandall^16^ had suggested that a seven-point scale anchored with the words *delighted* and *terrible* was more sensitive and less negatively skewed than a five-point satisfaction scale for quality of life assessment, probably because it allowed for a broader range of affective responses to QOL items.^17^

### Impact on life of CSP

Finally, two open-ended questions slightly modified from the previously published questions by Nickel et al.^14^ were included in the questionnaire. The first question was, “Describe the impact of the scrotal pain on your life,” and the second was, “Describe what changes you have made in your life because of the scrotal pain.” Two of the authors (OA and CW) reviewed the results to identify, by consensus, common themes.

## Results

The mean age of the men was 43 ± 12 (SD) years with a mean duration of CSP of 4.7 ± 5.95 years.

### Pain severity

In our sample of men, the pain was quite severe but intermittent (mean pain severity of 7.2 ± 2 on a ten-point numeric pain scale that affected men on average 40% ± 30% of the time). Constant background pain occurred in most men (126/131) with a pain level mean of 5.7 ± 2.3.

### Effect of CSP on work and normal activities

CSP significantly affects the day-to-day lives of many men. Work was affected, with 68/131 (51.90%) patients reporting decreased ability to work or the complete inability to work as a result of the CSP. The more severe the pain and the more frequent the severe pain episodes, the greater the reported level of interference with work, with only 6.6% (1/15) of men with reported pain levels of <5/10 reporting that the pain interfered “a lot” with the ability to work, compared to 51% (22/43) of those with pain levels of 9–10/10 (*P* < 0.01, Fisher’s exact test). If the men experienced the pain 80%–100% of the time, 57% (16/30) reported that the pain interfered “a lot” with the ability to work, whereas only 12% (7/57) of those reporting severe pain episodes <40% of the time noted the same effect on the ability to work (*P* < 0.01, chi-square test).

Independent of a perceived inability to work, 55/131 (42 %) patients reported that there was deterioration in their work performance (Table 1).

**Table 1.** Has there been a recent deterioration in your work performance?

Yes	55 (42%)
No	76 (58%)

Other activities were also affected: 93/131 (70.99%) patients reported decreased ability to perform normal daily activities, with the level of pain again being directly and significantly related to the perceived effect on normal activities. Of the 89 men with pain ≥ 7/10, 51% reported that the pain interfered “a lot” with normal activities, whereas only 10% of the 39 men with pain < 7 reported the same effect on normal activities (*P* < 0.01, chi-square test). Sexual activity was also affected, with 81/131 (61.83%) reporting decreased sexual activity and/or function. Again, there was a significantly higher impact on those with more severe pain, with 45/83 (54%) of men with pain levels of ≥7/10 reporting that the pain interfered “a lot” with sexual activity, whereas, conversely, only 13.5% (5/37) with reported pain < 7/10 noting that the CSP interfered “a lot” with sex (*P* < 0.01, chi-square test).

### Depressive symptoms

Depressive symptoms were common, with more than 31% of patients complaining of depressive symptoms more than half the days of the month (Table 2). The more severe the pain level, the higher the rates of depression, with 40% of men (35/88) with pain levels ≥ 7 noting feelings of depression more than half the time compared to 13% (5/38) of those with pain levels < 7 (*P* < 0.01, Fisher’s exact test). Nineteen out of 131 (14.50%) patients were bothered by little interest or pleasure in doing things more than half of the days, with 14.50% bothered “nearly every day” (Table 3). Men with more severe pain (pain scores ≥ 7) were significantly more likely to be bothered by feelings of little interest or pleasure more than half the time (35% [28/80] vs. 16% [6/38] for men with pain levels < 7; *P* < 0.01, chi-square test). Despite the high rates of depressive symptoms, current suicidal ideation was not reported by any of the men in this series.

**Table 2.** During the past month, have you often been bothered by feeling down, depressed, or hopeless?

	Pain level < 7	Pain level 7–10	Total
	*n* = 38	*n* = 88	*N* = 126
Not at all	22 (58%)	17 (19%)^a^	39 (31%)
Several days	11 (29%)	36 (41%)	47 (37%)
More than half the days	3 (7.9%)	15 (17%)	18 (14%)
Nearly every day	2 (5.2%)	20 (23%)^b^	22 (17%)

^a^Significantly different *P* < 0.01, chi-square test. ^b^Significantly different *P* < 0.01, Fisher’s exact test.

**Table 3.** During the past month, have you often been bothered by little interest or pleasure in doing things?

	Pain level < 7	Pain level 7–10	Total
	*n* = 38	*n* = 80	*N* = 118
Not at all	23 (60%)	22 (28%)^a^	45 (38%)
Several days	9 (24%)	30 (38%)	39 (33%)
More than half the days	3 (7.9%)	16 (20%)^b^	19 (16%)
Nearly every day	3 (7.9%)	12 (15%)^b^	15 (13%)

^a^Significantly different *P* < 0.01, chi-square test. ^b^Significantly different *P* < 0.01, Fisher’s exact test.

### Quality of life

The patients presented with an average QoL score of 5.91 ± SD 1.18, with 88 /131 (67.17%) patients responding with “unhappy” or “terrible” (Table 4). Feeling “terrible” was significantly more common in those with higher pain levels (≥7/10) in 47% (41/88) of the men compared to those with lower pain levels, with 8.1% (3/37) feeling “terrible” (*P* < 0.01, Fisher’s exact test). Even in those with only slightly different recorded pain levels (7–8 vs. 5–6), the frequency with which patients reported that they felt “terrible” was significantly higher among those with pain levels of 7–8/10 (33%; 15/45) compared to those with slightly lower pain levels of 5–6/10, where only 8% (2/25) reported that they feel “terrible” (*P* < 0.01, Fisher’s exact test).

**Table 4.** If you were to spend the rest of your life with your symptoms just the way they have been during the last month, how would you feel about that?

	Pain level < 7	Pain level 7–10	Total
	*n* = 37	*n* = 88	*N* = 125
Delighted	0	0	0
Pleased	1 (2.7%)	0	1 (0.8 %)
Mostly satisfied	3 (8.1%)	0	3 (2.4%)
Mixed	9 (24%)	5 (5.7%)	14 (11%)
Mostly dissatisfied	9 (24%)	13 (15%)	22 (18%)
Unhappy	12 (32%)	29 (33%)	41 (33%)
Terrible	3 (8.1%)	41 (47%)^a^	44 (35%)

^a^Significantly different *P* < 0.01, Fisher’s exact test.

### Impact of CSP on life

The impact of CSP was further explored through content analysis of the answers to the two open-ended questions. The answers were codified and separated into different content areas based on common themes.

Common themes that arose included the following:

1. The impact of CSP on their lives, with patients most commonly described effects on limiting activities (59%), difficulties with work (28%), and emotional effects (27%).
2. The severity of CSP, which varied among the patients, ranging from mild annoyance to devastating, where one patient described himself as “wheelchair bound” due to CSP.
3. Difficulty sleeping; that is, 13% of patients reported difficulty sleeping.

## Discussion

Quality of life measures have become a vital and often required part of health outcomes appraisal. For populations with chronic disease, measurement of QoL provides a meaningful way to determine the impact of health care when cure is not possible. Over the past 20 years, hundreds of instruments have been developed that purport to measure QoL.^18^ In our study, close to 70% of the patients with CSP reported feeling unhappy or terrible about their condition, with close to 50% of patients complaining of depressive symptoms.

This is the first study to use a standardized instrument to measure the QoL in men with CSP. Though any clinician treating men with CSP will likely perceive the significant reduction in QoL experienced by these men, this is the first study to quantify the QoL in this group.

We were also able to quantify the depressive symptoms, with the finding that close to 50% of the men reported depressive symptoms on a regular basis. Though it is unclear whether this depression was directly related to the chronic pain, adaption issues, or frustration, it is clear that clinicians should be aware of the psychological impact of CSP and ask questions to elicit information on depression. Depressive symptoms were particularly common among men with pain scores ≥ 7. Perhaps men presenting with pain scores ≥ 7 may benefit from further depression screening with validated scales such as the Beck Depression Inventory, which may provide opportunity for further psychiatric evaluation/intervention where it may not have been previously considered.^19^

Men with CSP frequently suffer from limitations in ability to work, social activities, and sexual functioning, any and all of which could contribute to lower QoL and depressive symptoms.

Though it should not be surprising that QoL, depressive scores, and limitations on activities are directly related to the pain levels reported, what surprised us was how dramatically different the men were with high or low pain scores. Most men with pain scores < 7 continued to live with generally a high QoL, with comparatively few reporting impacts on work, physical activities, or mood. The men who report pain scores ≥ 7, however, very frequently were unhappy or described their condition as being terrible. At the extreme end, the men described their condition as devastating, with some even being wheelchair bound due to the disability. These men with more severe pain also commonly reported depressive feelings on a regular basis and reduced pleasure-seeking behavior.

Within our population, there appeared to be two distinct groups, those with pain scores ≥ 7 and those with lower pain scores. The men with lower pain scores were usually able to continue with relatively normal activities and maintain good QoL, whereas the men with higher pain scores seemed to be dramatically affected by the CSP, with poor QoL, depressive symptoms, and reduced activities.

This study emphasizes the significantly reduced QoL, activity levels, and mood of men with CSP, particularly men experiencing high pain levels. Evaluating patients with chronic scrotal pain should include a psychosocial evaluation to include information on QoL, mood, and activity impairment.

A limitation of this study was the cross-sectional design of the study, which limits the ability to attribute the reduced QoL, mood, and activity levels found in men with CSP directly to the CSP. A longitudinal study capturing alterations in QoL, mood, and activity levels related to changes in pain levels with CSP would help to determine whether the CSP led to the symptoms described above. The fact that this is a single-center study limits the generalizability of this study. Multicenter studies are required to more completely understand this debilitating condition.

## Conclusion

In our study, CSP was associated with an extremely poor quality of life, with high rates of depressive symptoms and significant limitations in work, social, and sexual activities. The association was most profound for men with high pain levels, whereas men with lower pain levels continued to have good QoL, mood, and activity levels.
